# High-Throughput Screening of Australian Marine Organism Extracts for Bioactive Molecules Affecting the Cellular Storage of Neutral Lipids

**DOI:** 10.1371/journal.pone.0022868

**Published:** 2011-08-08

**Authors:** James Rae, Frank Fontaine, Angela A. Salim, Harriet P. Lo, Robert J. Capon, Robert G. Parton, Sally Martin

**Affiliations:** 1 Division of Molecular Cell Biology, Institute for Molecular Bioscience, The University of Queensland, Brisbane, Queensland, Australia; 2 Division of Chemical and Structural Biology, Institute for Molecular Bioscience, The University of Queensland, Brisbane, Queensland, Australia; Chinese University of Hong Kong, Hong Kong

## Abstract

Mammalian cells store excess fatty acids as neutral lipids in specialised organelles called lipid droplets (LDs). Using a simple cell-based assay and open-source software we established a high throughput screen for LD formation in A431 cells in order to identify small bioactive molecules affecting lipid storage. Screening an *n*-butanol extract library from Australian marine organisms we identified 114 extracts that produced either an increase or a decrease in LD formation in fatty acid-treated A431 cells with varying degrees of cytotoxicity. We selected for further analysis a non-cytotoxic extract derived from the genus *Spongia (Heterofibria)*. Solvent partitioning, HPLC fractionation and spectroscopic analysis (NMR, MS) identified a family of related molecules within this extract with unique structural features, a subset of which reduced LD formation. We selected one of these molecules, heterofibrin A1, for more detailed cellular analysis. Inhibition of LD biogenesis by heterofibrin A1 was observed in both A431 cells and AML12 hepatocytes. The activity of heterofibrin A1 was dose dependent with 20 µM inhibiting LD formation and triglyceride accumulation by ∼50% in the presence of 50 µM oleic acid. Using a fluorescent fatty acid analogue we found that heterofibrin A1 significantly reduces the intracellular accumulation of fatty acids and results in the formation of distinct fatty acid metabolites in both cultured cells and in embryos of the zebrafish *Danio rerio*. In summary we have shown using readily accessible software and a relatively simple assay system that we can identify and isolate bioactive molecules from marine extracts, which affect the formation of LDs and the metabolism of fatty acids both *in vitro* and *in vivo*.

## Introduction

Fatty acids are essential components of all organisms, serving as precursors for phospholipid synthesis, substrates for energy production and acting as signalling molecules. Fatty acids are stored as neutral lipids, predominantly triglycerides (TGs) and cholesteryl esters, in intracellular organelles termed lipid droplets (LDs) (reviewed in [1], [2]). Long considered inert lipid storage depots, LDs are now recognised as dynamic organelles with a complex regulation. Despite the clear importance of LDs both physiologically and pathologically very little is known about the mechanisms underlying their biogenesis, maturation and catabolism. LDs play a crucial role in maintaining the cellular levels of lipids and cholesterol by regulating the interplay between storage (as lipid esters), hydrolysis (lipolysis) and trafficking [2], [3]. Physiologically abnormal lipid accumulation is associated with a wide range of pathophysiological conditions including obesity, atherosclerosis, neutral lipid storage disorders and lipodystrophies. Despite the ubiquitous nature of LDs our understanding of their function and regulation in most cell types is limited, and there are currently few chemical tools available to aid our study of either the LDs themselves or the intracellular trafficking and targeting of fatty acids. The identification of small bioactive molecules that can be used to manipulate triglyceride storage in cells will be invaluable in the search for potential therapeutic agents and therapeutic targets, and to develop small molecule reagents with applications to basic research.

Historically, many small molecule inhibitors have been identified following extraction of metabolites from natural sources. Over recent years a number of small molecules isolated from natural sources have been shown to affect LD accumulation in macrophage cells or to affect the accumulation neutral lipids during the differentiation of model adipocyte cell lines [4]–[11], both cell types in which LD formation is uniquely and specifically regulated. As LDs are ubiquitous organelles we undertook to develop a screen for small bioactive molecules modulating the universal aspects of lipid storage. The presence or absence of LDs in cells maintained in tissue culture can be readily manipulated by altering the growth conditions of the cells. Growth in lipid-depleted serum results in the loss of LDs and neutral lipids. In contrast growth in medium containing elevated levels of free fatty acids such as oleic acid results in the storage of neutral lipid and the *de novo* formation of LDs. Both the formation and the loss of LDs in cells can be observed microscopically using dyes that fluoresce when present in neutral lipid environments [12]. We have used these properties of lipid droplets in order to screen a library of natural extracts derived from Australian marine organisms for molecules with the ability to modulate LD formation. We established a semi-automated quantitative cell-based confocal microscopy assay to test over 2000 extracts for effects on LD formation and have identified a number of distinct extracts containing potent modulators of lipid droplet metabolism. One of these extracts was further analysed and the specific bioactive molecular components isolated and functionally analysed both *in vitro* and *in vivo*. These data confirmed both the suitability of the screening approach and the presence within natural product extracts of molecules with the potential for development as therapeutic and/or research agents.

## Materials and Methods

### Ethics Statement

Zebrafish studies were carried out according to NHMRC (Australia) guidelines and Animal Ethics Approval IMB/438/08/NHMRC (NF) to Prof Parton.

### Cell culture and reagents

A431 cells (American Type Culture Collection, Rockville, MD) were maintained in Dulbecco's modified Eagle's high glucose medium (DMEM) supplemented with 10% (v/v) fetal calf serum (FCS) and 2 mM L-glutamine. AML12 hepatocytes were maintained in Dulbecco's modified Eagle's nutrient mixture F12 medium with 10% (v/v) heat-inactivated serum supreme, 2 mM L-glutamine, 1 mM sodium pyruvate and 0.1 mM non-essential amino acids. Oleic acid (Calbiochem) and Bodipy-558/568 C_12_ (Invitrogen) were prepared as 50 mM stock solutions in ethanol. Bodipy493/503 (Invitrogen) was prepared as a saturated stock solution in ethanol. 4′,6-diamidino-2-phenylindole (DAPI) was prepared as a 1 mg/mL stock in water. A mixture of mono-, di-, and triglyceride standards were used for thin-layer chromatography (Sigma cat # 1787-1AMP). Lactate dehydrogenase levels were measured using the Cytotoxicity Detection Kit (Roche Diagnostics, Germany). [9,10-3H(N)]-Oleic acid was obtained from PerkinElmer (Browns Plains, Australia). All other reagents were from Sigma unless stated otherwise.

### Delipidated fetal calf serum (DL-FCS)

FCS was delipidated using organic solvent extraction [13]. FCS, diisopropyl ether and *n*-butanol (5∶4∶2) were homogenised using a magnetic stirrer and centrifuged (1700 rpm for 15 min) to induce phase separation. The lower aqueous phase was collected and re-extracted with diisopropyl ether. Residual diisopropyl ether was removed under a stream of nitrogen gas. DL-FCS was dialysed (10,000 MWCO) into PBS, sterilised through 0.22 µm filter units and stored at −20°C.

### Primary assay protocol

A431 cells were plated at a density of 15,000 cells/well (100 µL) onto black wall 96-well imaging plates (BD Falcon) in DMEM supplemented with 10% (v/v) DL-FCS, 2 mM L-glutamine, penicillin (100 units/mL) and streptomycin (100 µg/mL) (hereafter referred to as assay media) and allowed to adhere for 3 h. *n*-Butanol extracts or sub-fractions were dried onto 96-well plates and re-suspended in assay medium prior to horizontal transfer (100 µL/well) into duplicate wells of cells on the imaging plate to achieve the desired final extract concentration. Control wells received an equivalent volume of either assay media or triacsin C. Following incubation for 15–18 h cells were supplemented with 50 µM oleic acid for a further 8 h. At the end of the assay cells were washed twice in PBS prior to fixation in 4% paraformaldehyde (PFA)/PBS for 20 min. PFA was removed and the wells quenched with 50 mM NH_4_Cl/PBS, 30 min. Cells were subsequently co-stained with Bodipy 493/503 (0.5% v/v of saturated solution at 20°C) and DAPI (0.1 µg/mL) for 30 min. Finally the cells were washed twice with PBS and stored in 200 µL PBS at 4°C.

### Secondary assay protocols

Purified heterofibrins were prepared at 50 mM in DMSO and diluted in assay media to achieve the final concentrations shown in the results section. To measure LD formation, cells were treated with purified heterofibrins for 15–18 h prior to supplementation of the media with 50 µM oleic acid for 8 h and processing as described above for the primary assay. To measure the uptake and metabolism of [^3^H] oleic acid or Bodipy-558/568 C_12_ cells were incubated with the relevant heterofibrins overnight as described above. Cells were subsequently supplemented with either 50 µM oleic acid containing 5 µCi [^3^H] oleic acid or with 50 µM Bodipy-558/568 C_12_ for various times as shown in the results sections. Cells were subsequently processed for thin-layer chromatography as detailed below, or fixed in 4% PFA/PBS for fluorescence microscopy.

### Image acquisition and analysis

Plates were imaged using a BD Pathway 855 microscope (BD Biosciences) running Attovision 1.5/1.6 software. To measure cell number (as a surrogate for cytotoxicity) a 1×2 montage epifluorescent DAPI image was captured using an Olympus UPlanSApo 10x/0,40 objective. For lipid droplet analysis single or 1×2 montage confocal images were acquired for DAPI (single optical slice) and Bodipy493/503 (collapsed Z stack) using an Olympus VApo/340 40X/0,90 objective. Raw data images were saved as 16 bit Tiff files for export. Image analysis was undertaken using CellProfiler (1.0.5122) open-source software [14] (The Broad Institute, http://www.cellprofiler.org *)*. The image analysis pipelines are detailed in supplementary data (Tables S1, S2 and S3). Where appropriate, statistical significance was determined using a two-tailed unpaired Student's t-test.

### Natural product extract partitioning and fractionation

Raw specimen samples were stored in ethanol at −20°C. A detailed description of the partitioning, fractionation and subsequent structural analysis of specimen extracts is described in [15]. Briefly, samples in ethanol were dried by rotary evaporation and used to prepare *n*-butanol partitions. The primary assay was performed using the *n*-butanol extracts. Lead *n*-butanol extracts containing bioactivity were further resolved by sequential partitioning into hexane, dichloromethane or methanol. Sub-fractions that retained the bioactivity of the parent extract were subjected to chemical profiling using HPLC-DAD-MS and ^1^H NMR and further fractionation by preparative HPLC to isolate bioactive molecules. Detailed structural analysis was achieved using 1D and 2D NMR and HRMS.

### Lipid analysis by thin layer chromatography

A431 cells were treated with *n*-butanol extracts or purified inhibitors as described in the results sections. Cells were subsequently washed with PBS, trypsinised and the cell pellet resuspended in 0.25 mL PBS. Neutral lipids were extracted using 1 mL of methanol:chloroform (2∶1) and vortexing. Following drying under N_2_ and concentration, neutral lipids were redissolved in methanol:chloroform (2∶1). Lipids were loaded onto HP-TLC silica plates and developed in hexane/diethylether/acetic acid (70∶30∶1). Radiolabelled samples were developed by autoradiography. After development plates were sprayed with a primuline solution (50 µg/mL in acetone:water, 80∶20) and visualised by UV.

### *In vivo* analysis of heterofibrin activity in zebrafish

Wild type zebrafish were raised as described previously [16]. Zebrafish embryos (4–6 dpf) were incubated in E3 embryo media (5 mM NaCl, 0.17 mM KCl, 0.33 mM CaCl_2_, 0.33 mM MgSO_4_) in a 12-well plate in the presence or absence of 10 µM heterofibrin A1 or 0.5 µM triacsin C for 2 h prior to the addition of Bodipy558/568 C_12_ for 6 h. Embryos were immobilised in E3 containing tricaine (0.2% 3-aminobenzoic acid ethyl ester), incubated on ice for 30 min, then either fixed in 4% paraformaldehyde in PBS overnight or homogenised in 200 µL E3 for lipid extraction. Fixed embryos were mounted on concave slides and imaged using an Olympus SZX-12 stereodissecting microscope with a DP-70 12MP colour camera. Lipid extraction for TLC was performed as described above.

## Results

### An automated high throughput screen for LD formation in A431 fibroblasts

**Figure 1:**
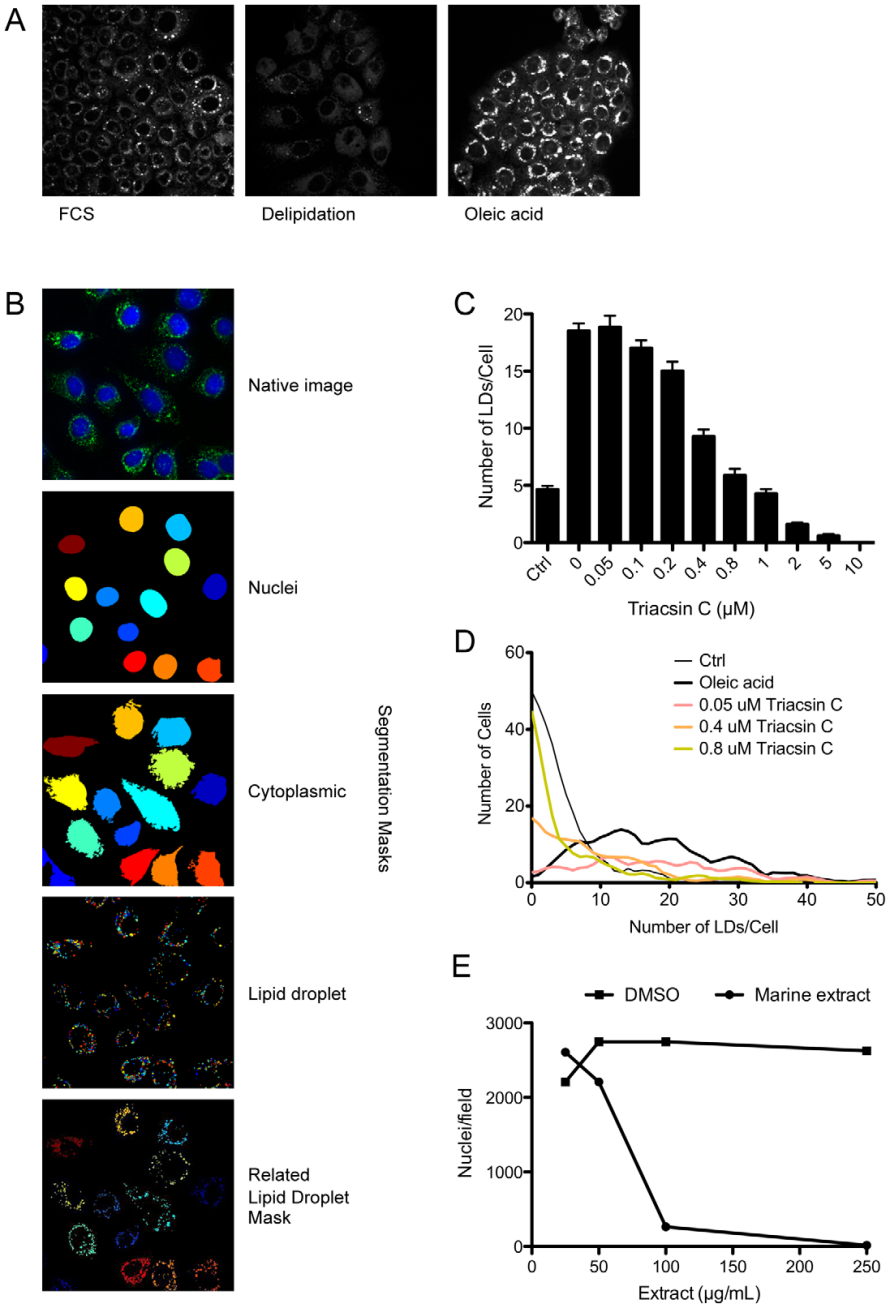
Assay design and validation. (A) A431 cells were maintained for 18 h in media containing either 10% FCS (FCS), 10% delipidated FCS (delipidation) or 10% delipidated FCS supplemented with 50 µM oleic acid (oleic acid). Cells were fixed and stained for LDs using Bodipy493/503. (B) A431 cells were maintained for 8 h in 10% delipidated FCS supplemented with 50 µM oleic acid. Following fixation cells were stained for LDs using Bodipy493/503 (green) and nuclei (blue). Native images were obtained using a BD Pathway automated confocal microscope. CellProfiler software was used to generate segmentation masks to identify individual nuclei, cytoplasmic area and LDs, and to subsequently generate a related LD mask, using the analysis pipeline detailed in Materials and Methods, and Tables S1 and S2. (C, D) The ability of the automated analysis pipeline to quantify LD formation was examined using triacsin C, a potent inhibitor of TG synthesis. A431 cells were maintained overnight in delipidated serum ± varying concentrations of triacsin C. Cells were subsequently supplemented with 50 µM oleic acid for 8 h. Images of fixed, labelled cells were analysed using the CellProfiler analysis pipeline to determine both the mean number of LDs/cell ((C) mean ± sem, n>145 cells) or the frequency distribution of cells containing a specific number of LDs (D). Ctrl indicates cells incubated in delipidated serum only. (E) A431 cells were incubated with varying concentrations of inactivated generic *n*-butanol marine extract for 18 h. Cells were fixed, the nuclei stained using DAPI and the number of cells per field quantified.

In order to establish a cell-based high throughput screen for LD formation several fibroblast cells lines were assessed for their applicability for automated analysis. A431 human epidermoid carcinoma cells were found to generate LDs of a consistent size and number in response to 50 µM oleic acid and to lose endogenous LDs when cultured in delipidated serum (Fig. 1A) and were subsequently selected as the optimal cell line for assay development and for the primary screen. Cells were seeded in 96-well plates and grown in medium containing delipidated serum overnight, to establish conditions of minimal LD number. Cells were subsequently supplemented with 50 µM oleic acid for 8 h to promote LD biogenesis. After fixation the cells were co-stained with Bodipy493/503 and DAPI, to identify LDs and nuclei respectively and subsequently imaged using a BD pathway automated confocal microscope. Nuclei were detected using epifluorescence microscopy and the number of nuclei per field used as a surrogate read-out in subsequent analyses for cytotoxicity. The LDs were detected using a confocal z-series, which was subsequently rendered into 2-dimensions. The number of LDs generated per cell was analysed using CellProfiler (Broad Institute Imaging Platform [14]) and the data reported as the average number of LDs per cell (Fig. 1B). The image analysis protocol is detailed in Materials and Methods and the CellProfiler analysis pipeline is available as supplementary data (Tables S1 and S2). Maintaining A431 cells in delipidated FBS overnight effectively reduced the number of LDs to <5 LDs/cell and subsequent treatment with 50 µM oleic acid for 8 h induced an ∼10-fold increase in the average number of LDs per cell.

In order to establish an assay window against which to measure the relative effect of candidate extracts on LD formation we used triacsin C, a potent inhibitor of long-chain acyl-CoA synthase enzymes [17]. By inhibiting the formation of acyl-CoAs, triacsin C prevents neutral lipid synthesis and LD formation [18]. Addition of triacsin C overnight was found to induce a dose-dependent reduction in the average number of LDs formed per cell during the subsequent 8 h treatment with 50 µM oleic acid (Fig. 1C). This result confirmed the ability of the image analysis pipeline to reliably quantify LDs and identified the optimal concentration of triacsin C (1 µM) used in subsequent experiments. The image analysis pipeline could also be used to determine the distribution curve of LDs per cell (Fig. 1D). Using this analysis we could show that the number of LDs per cell increased with oleic acid treatment (right shift), and showed a dose-dependent shift back to the left with increasing concentrations of triacsin C.

To examine the underlying cytotoxicity of marine extracts on A431 cells, we performed growth curves with an inactivated extract (exposed to ambient light and temperature for 72 h to minimize active components). A concentration of 50 µg/mL *n*-butanol extract was selected as the optimal non-toxic concentration (Fig. 1E). In order to internally control for inter-plate variability, both triacsin C (1 µM) and inactive extract (50 µg/mL) were routinely included on every assay plate. These conditions were adopted as the standard protocol for the subsequent primary assay.

### Primary screen of a natural extract library from Australian marine organisms

**Figure 2:**
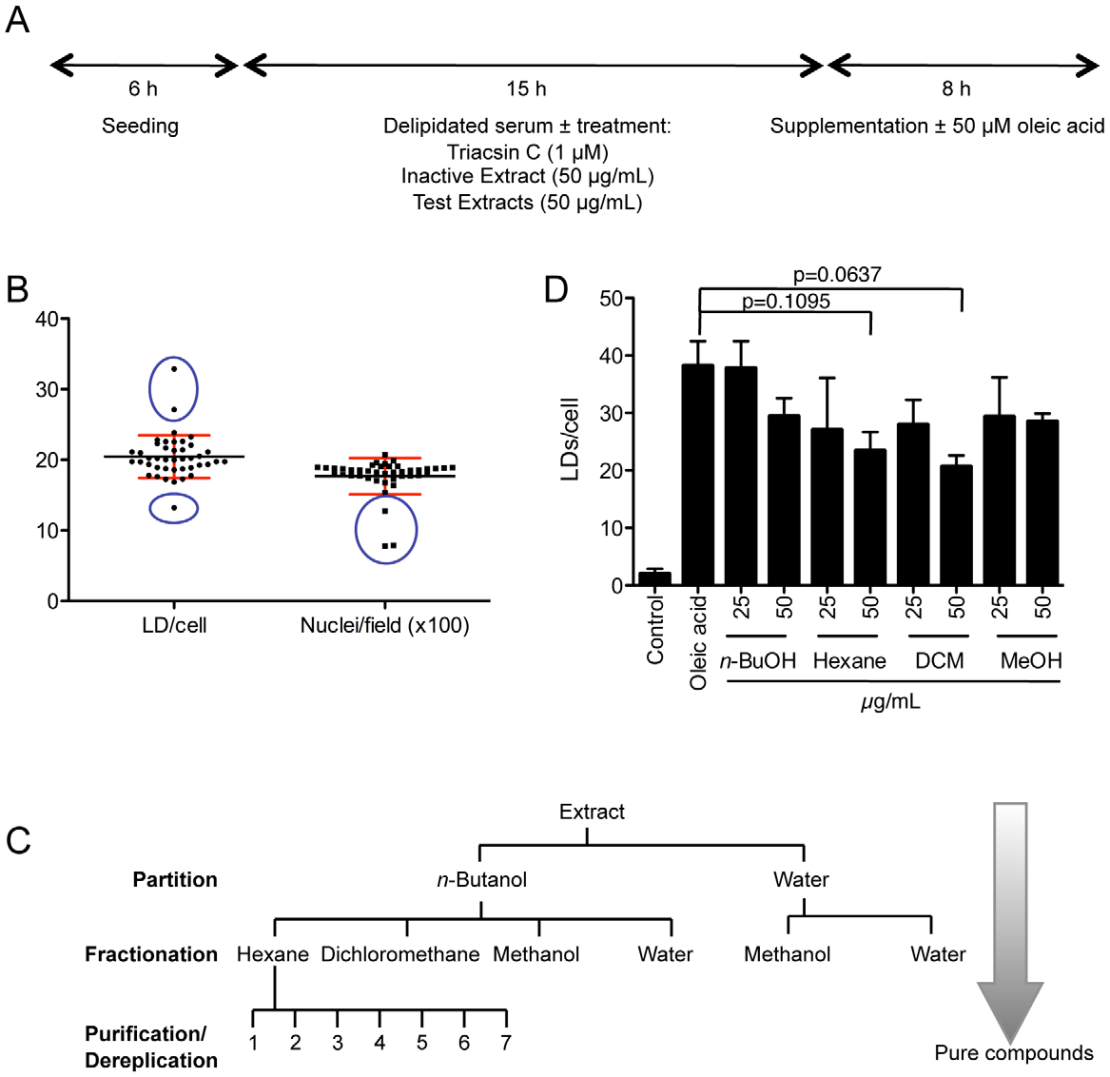
Extract analysis and identification of heterofibrin A1. (A) LD biogenesis assay design. A431 cells were seeded for 6 h in growth medium then transferred into delipidated medium supplemented with control reagents or experimental extracts. Following incubation for 15 h cells were further supplemented with oleic acid for 8 h prior to fixation and processing. (B) Results obtained from a typical 96-well plate in the primary screen. A431 cells were incubated with *n*-butanol marine extracts at 50 µg/mL overnight prior to supplementation with 50 µM oleic acid for 8 h. Cells were subsequently fixed, stained for cell nuclei (DAPI) and LDs (Bodipy493/503). The average number of LDs/cell and the average number of nuclei/field were quantified. Each point represents the average of duplicate wells treated with a single extract. Results are shown for 42 randomly chosen extracts equivalent to one 96-well plate, and error bars represent the SD within the plate. Circled areas delineate marine extracts that change either the number of LDs per cell or nuclei per field relative to the normal range. (C) Schematic representation of the marine extracts processing protocol. Extract were initially partitioned between *n*-butanol and water. *n*-Butanol partitions identified as containing putative bioactive molecules were sequentially fractionated into hexane, dichloromethane or methanol, and individual fractions analysed for their effects on LD formation. Final resolution of the active components was achieved using HPLC-DAD-MS and NMR. (D) An *n*-butanol extract derived from a species of *Spongia (Heterofibria)* (designation CMB-03399) was fractionated as described in (C) and the effect of derived sub-fractions on LD formation analysed at different concentrations (n = 2 separate experiments, ± SD). DCM = dichloromethane, MeOH = methanol, *n*-but = *n*-butanol.

To identify specific bioactive molecules affecting LD formation we followed the partitioning and fractionation protocol detailed in Fig. 2C. We hypothesised that molecules active in the lipid metabolism pathways would partition predominantly into organic fractions. We therefore performed a primary screen of 2184 *n*-butanol extracts derived from marine organisms collected across Australian temperate and Antarctic ecosystems. Each extract was tested in duplicate and both the mean number of LDs per cell and the mean number of nuclei per field, averaged between the two wells. A typical screening plate contained 42 randomly selected marine extracts in addition to an inactive extract control (50 µg/mL) together with 1 µM triacsin C to account for inter-plate variability and matrix effects. For each well the number of LDs/cell and the number of nuclei/field was quantified. A typical dataset derived from 42 randomly chosen extracts (1 plate) is shown in Fig. 2B. For each individual dataset the average number of LDs/cell ± SD was determined and an extract was considered to contain potential bioactivity if the number of LDs/cell fell outside the normal range across the plate. Potentially bioactive extracts were verified by visually scoring to ensure the accuracy of the results. From a primary screen of 2184 extracts at a concentration of 50 µg/mL, a total of 87 extracts (4%) resulted in an increased number of LDs/cell, while 27 extracts (1%) resulted in a decrease in LD number. These results suggest that within the screened extracts there are bioactive molecules that can either augment or reduce LD accumulation. Similarly cytotoxicity was assumed when the number of nuclei/field fell outside the normal range (±SD) across the plate. Only a small proportion (3%) of the extracts were found to affect cell number. Interestingly a high proportion of extracts potentially exhibiting cytotoxicity also resulted in an increase in the number of LDs detected per cell (35%). In contrast, only 5% of extracts which were considered cytotoxic also resulted in a decrease in LD number. Whether this reflects a biological process such as a compensatory or protective adaptation to the cytotoxicity of the extract is currently unknown.

A subset of *n*-butanol extracts containing inhibitory bioactivity were sub-fractionated into hexane, dichloromethane or methanol, and re-screened across a range of concentrations between 1-50 µg/mL. The effect of the sub-fractions on LD formation was analysed as above. After rescreening we recovered bioactivity in one or more sub-fractions derived from nine *n*-butanol extracts (Table 1). In most cases the highest levels of bioactivity were identified in the hexane and/or dichloromethane fractions, consistent with enrichment in organic inhibitors. Fig. 2D shows the distribution of bioactivity in sub-fractions obtained from an extract derived from a species of *Spongia (Heterofibria)* sp. (designation CMB-03399, Table 1). Both the original *n*-butanol extract of *Spongia (Heterofibria)* sp. (Table 1) and the sub-fractions were found to inhibit LD formation (Fig. 2D) without any detectable cytotoxicity (data not shown). We therefore selected this extract for further analysis. As the hexane fraction contained the highest level of bioactivity this fraction was subjected to further purification.

**Table 1:** Screening of sub-fractions derived from a subset of inhibitory *n*-butanol extracts.

Extract ID	*n*-Butanol extract effect	Sub-fractions with confirmed activity	Cytotoxicity of sub-fractions
CMB-03399	Inhibition	Hexane, DCM	nd
CMB-01523	Inhibition	Hexane, DCM	nd
CMB-01550	Inhibition	Hexane, DCM	nd
CMB-02242	Inhibition	Hexane, DCM, Methanol	nd
CMB-01081	Inhibition	Hexane, DCM	>1 µg/mL
CMB-03450	Inhibition	DCM	>1 µg/mL
CMB-02727	Inhibition	DCM, Methanol	DCM>1 µg/mL
CMB-03361	Inhibition	Hexane, DCM	Hexane>25 µg/mL DCM>5 µg/mL
CMB-01180	Augmentation	Hexane, DCM	>5 µg/mL

nd = no detectable cytotoxicity at 50 µg/mL.

### Identification of heterofibrin A1 as a bioactive molecule in *Spongia (Heterofibria)* sp. extract

**Figure 3:**
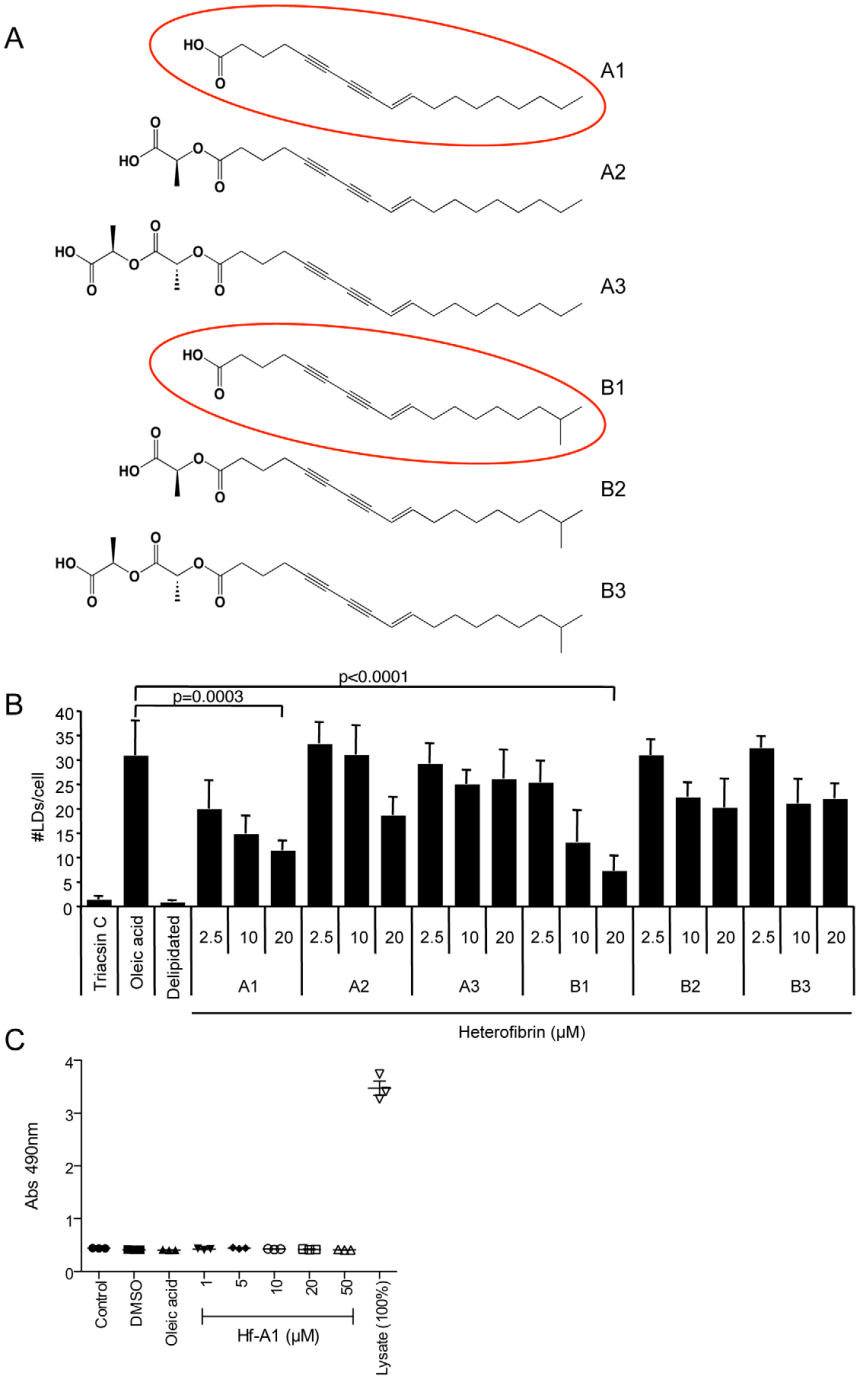
Effect of heterofibrins on LD formation. (A) A family of related diyne-ene fatty acids designated heterofibrin A1–A3 and B1–B3 were isolated from the hexane fraction of *Spongia (Heterofibria)* sp. A detailed structural analysis of the identification of the heterofibrin family of molecules is described in [15]. (B) The activity of six heterofibrins on LD formation was analysed in A431 cells. Each heterofibrin was analysed across a range of concentrations. The highest levels of activity were associated with heterofibrins A1 and B1 (mean ± sem, n = 4 separate experiments). (C) The effect of heterofibrin A1 on cell viability was analysed by measuring LDH release. Cells were incubated for 16 h in control medium, DMSO, 50 µM oleic acid or varying concentrations of heterofibrin A1 (Hf-A1). At the end of the incubation period LDH activity in either the media or in solubilised whole cell lysates were measured according the manufacturers instructions (mean ± sem, n = 3 separate experiments).

To resolve individual bioactive molecules from the hexane fraction of *Spongia (Heterofibria)* sp. we used HPLC-DAD-MS and NMR spectroscopic methods. This analysis resulted in the isolation and identification of six novel acetylenic compounds, heterofibrins A1–A3 and B1–B3, containing the rare diyne-ene moiety (Fig. 3A). The chemical identification methodology and structural analysis of these molecules is described in detail in our associated publication [15]. The ability of each of the heterofibrins to inhibit LD biogenesis was analysed across a range of concentrations (Fig. 3B). Two members of the heterofibrin family of molecules, heterofibrins A1 and B1 were found to be most effective at inhibiting LD formation. In each case the inhibition of LD formation was dose-dependent, with a ∼60% inhibition observed at a concentration of 20 µM in the presence of 50 µM oleic acid. As the activities of heterofibrin A1 and B1 were similar, we focussed on heterofibrin A1, a more abundant molecule, in subsequent analyses. The effect of heterofibrin A1 on cell viability was determined over a range of concentrations by measuring the release of lactate dehydrogenase (LDH) (Fig. 3C). Heterofibrin A1 was found to be non-cytotoxic at concentrations up to 50 µM (the maximum tested) consistent with our previous finding that none of the heterofibrin molecules were cytotoxic at a concentration of 30 µM, determined using an MTT assay [15].

**Figure 4:**
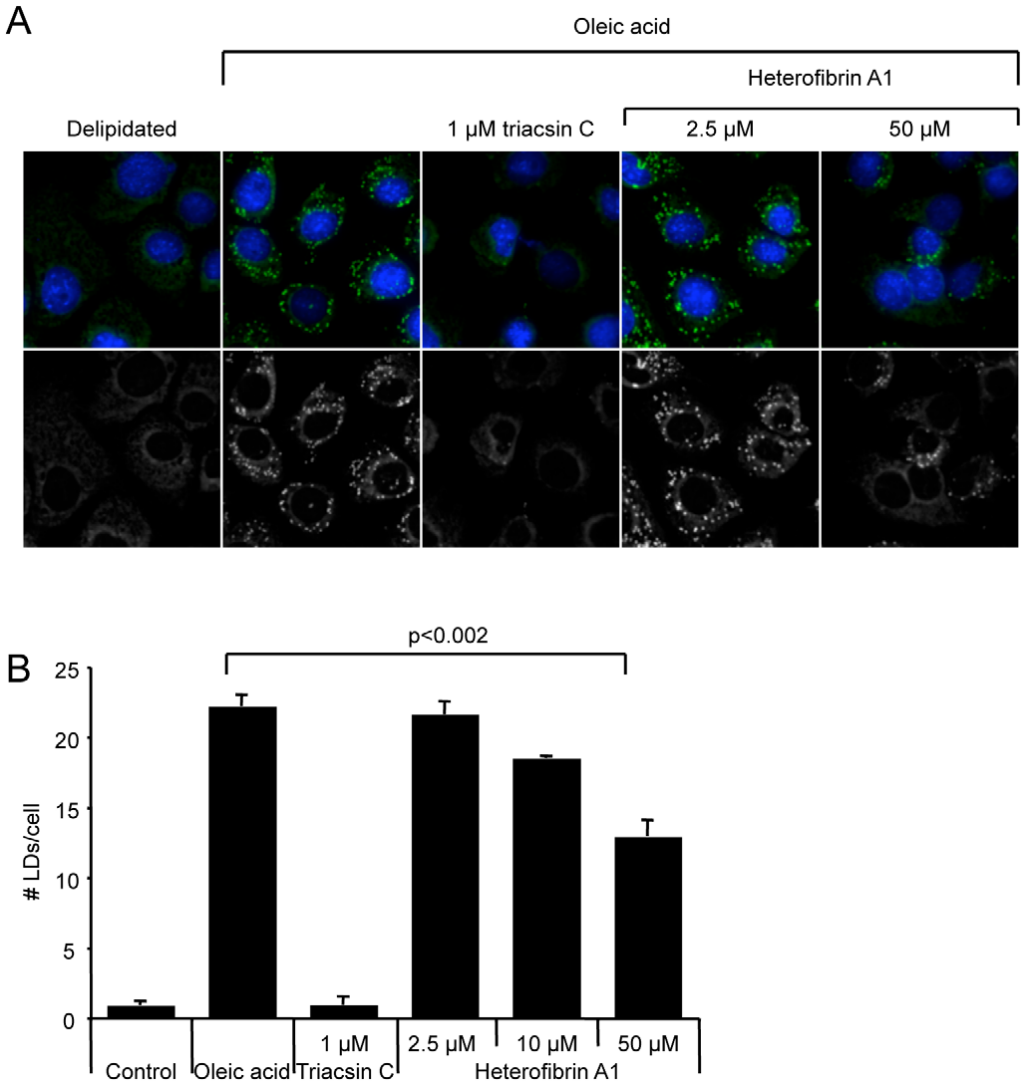
Heterofibrin A1 inhibition of LD biogenesis in AML12 hepatocytes. (A) AML12 hepatocytes were incubated overnight in delipidated FCS supplemented with either with 1 µM triacsin C or different concentrations of heterofibrin A1. Cells were subsequently treated with 50 µM oleic acid for 8 h. Control cells were incubated in delipidated FCS only. Cells were fixed and labelled for lipid droplets (Bodipy493/503, green) and nuclei (DAPI, blue). (B) Images of the fixed, labelled cells were analysed using the CellProfiler analysis pipeline and the number of LDs/cell quantified (mean ± sem, n = 3 separate experiments).

To confirm that the activity of heterofibrin A1 on LD formation was not restricted to A431 cells, and therefore the amenity of the screening process to identifying bioactive molecules with more widespread applications, we examined the activity of heterofibrin A1 on AML12 hepatocytes (Fig. 4). We selected a hepatocyte cell line as a number of pathophysiological conditions and diseases are associated with the abnormal accumulation of LDs in this cell type, including the development of non-alcoholic steatohepatitis [19] and assembly of the Hepatitis C virus [20]. AML12 cells were incubated in varying concentrations of heterofibrin A1 or with 1 µM triacsin C, and LD formation in response to 50 µM oleic acid analysed by confocal microscopy using the image analysis parameters described above. Consistent with the inhibitory effect of heterofibrin A1 on LD formation in A431 cells, there was also a significant inhibition of LD formation in AML12 hepatocytes. These data suggest that heterofibrin A1 affects a conserved element(s) in the LD biogenesis pathway.

### Functional analysis of heterofibrin A1 activity in A431 cells

**Figure 5:**
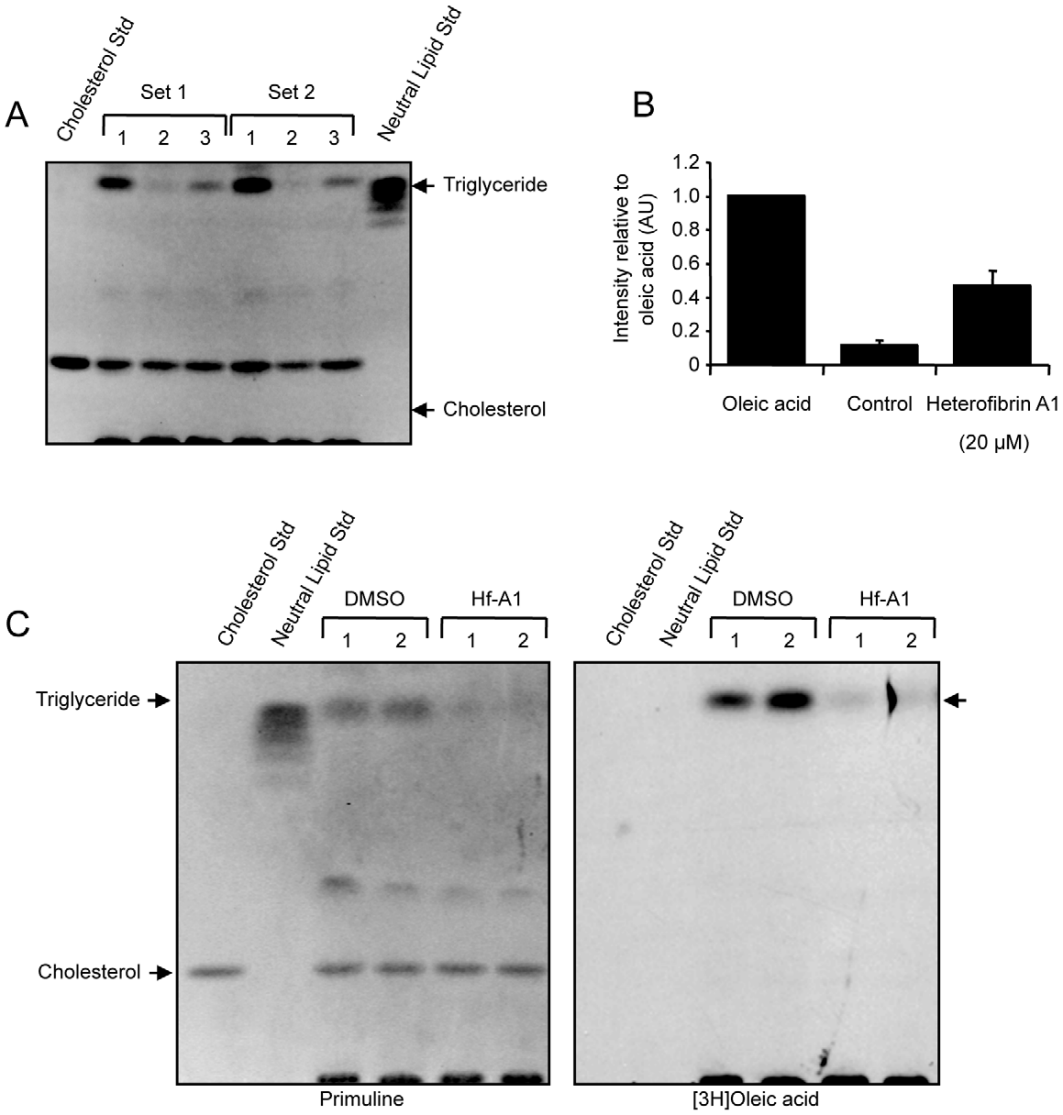
Quantification of triglyceride in A431 cells. (A) A431 cells were incubated as follows: (1) delipidated FCS 16 h, 50 µM oleic acid 8 h, (2) delipidated FCS 24 h, (3) delipidated FCS containing 20 µM heterofibrin A1 16 h, 50 µM oleic acid 8 h. Neutral lipids were extracted and resolved by TLC. Results are shown for two independent sets of cells. Neutral Lipid Std = 45 µg lipid standard, Cholesterol Std = 10 µg free cholesterol. (B) The accumulation of TG was quantified using Image J. Results shown are relative to oleic acid (mean ± sem, n = 4 separate experiments). (C) A431 cells were incubated in delipidated FCS containing either 20 µM heterofibrin A1 or DMSO for 16 h. Cells were subsequently supplemented with 50 µM oleic acid containing 5 µCi [3H] oleic acid for 6 h. Neutral lipids were extracted and resolved by TLC. Results are shown for two independent sets of cells. Neutral Lipid Std = 45 µg lipid standard, Cholesterol Std = 10 µg free cholesterol.

In order to determine whether heterofibrin A1 directly affected the biogenesis of LDs in response to oleic acid or the synthesis of triacylglycerol (TG) we analysed TG accumulation using thin-layer chromatography (TLC). A431 cells were incubated in delipidated serum overnight in the presence or absence of 20 µM heterofibrin A1. Cells were subsequently supplemented with 50 µM oleic acid for 8 h, total neutral lipids extracted and the amount of TG quantified following TLC (Fig. 5A). Oleic acid induced a marked increase in the cellular TG content relative to delipidated serum. Pre-incubation with heterofibrin A1 significantly abrogated the accumulation of TG in response to oleic acid treatment. Quantitation of the images demonstrated a ∼50% decrease in neutral lipid levels relative to oleic acid alone (Fig. 5B), consistent with the decrease in the number of LDs/cell shown in Fig. 3B, suggesting that the reduction in LD number can be attributed to a decrease in neutral lipid levels. To confirm an inhibition of TG synthesis we examined the metabolism of exogenous [3H] oleic acid (Fig. 5C). Consistent with the decrease in total TG levels we found that pre-treatment of A431 cells with heterofibrin A1 almost completely blocked the formation of TG. Furthermore, we observed no accumulation of intermediates on the TG biosynthetic pathway or radiolabelled phospholipids. From these data we hypothesise that the target(s) of heterofibrin A1 are likely to be involved in the uptake or early processing of fatty acids, rather than the TG biosynthetic pathways or directly in the formation of LDs.

### Effect of heterofibrin A1 on fluorescent fatty acid uptake and metabolism in cultured cells

**Figure 6:**
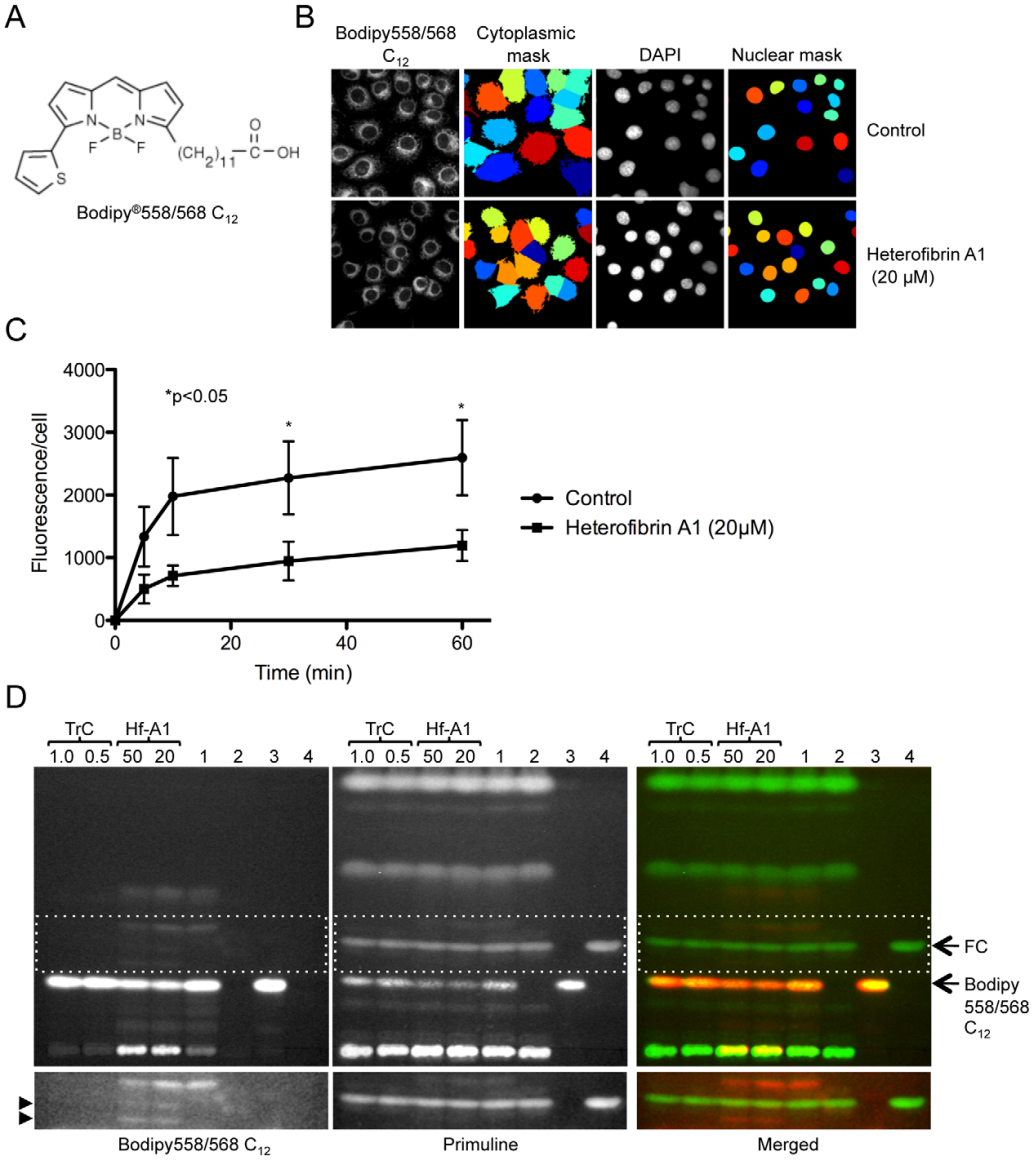
Fluorescent fatty acid uptake and metabolism in A431 cells. (A) Structure of Bodipy®558/568 C_12_ (Invitrogen). (B) A431 cells were incubated in delipidated FCS (control) or 20 µM heterofibrin A1 overnight. Cells were subsequently incubated with 50 µM Bodipy558/568 C_12_ for 30 min, fixed and cell nuclei labelled using DAPI. The accumulation of fatty acid within the cytoplasm of the cell was determined by Bodipy558/568 C_12_ fluorescence. Cytoplasmic and nuclear imaging masks were generated using CellProfiler and used to determine the average cytoplasmic intensity of Bodipy558/568 C_12_ fluorescence per cell. (C) The fluorescence intensity of cytoplasmic Bodipy558/568 C_12_ was quantified at various times over a 60 min period (mean ± sem, n = 4 expts). (D) A431 cells were incubated overnight in delipidated FCS, heterofibrin A1 (Hf-A1, 20–50 µM) or triacsin C (TrC, 0.5–1 µM), then supplemented with 50 µM Bodipy558/568 C_12_ for 2 h. Neutral lipids were extracted and resolved by TLC. Bodipy558/568 C_12_ was detected using its intrinsic fluorescence and total lipids detected using primuline. Lanes are as follows: (1) Cells incubated in 50 µM Bodipy558/568 C_12_ only for 2 h, (2) Control cells incubated in delipidated FCS only, (3) Bodipy558/568 C_12_, 24 µg, (4) cholesterol standard, 10 µg. Arrows indicate free cholesterol (FC) and free Bodipy558/568 C_12_. Arrowheads indicate Bodipy558/568 C_12_ metabolites detected in the heterofibrin A1 treated cells only. Lower panels highlight regions of the TLC plate incorporating novel metabolites.

To investigate further the nature of the cellular targets of heterofibrin A1 we examined both the influx and the metabolism of a fluorescently tagged fatty acid analogue, Bodipy558/568 C_12_ (Fig. 6A). A431 cells were incubated overnight in delipidated serum ±20 µM heterofibrin A1. The uptake of fatty acids into the cells was subsequently measured by the addition of 50 µM Bodipy558/568 C_12_ for various times between 0–60 min. Cells were fixed and the nuclei labelled using DAPI. We adapted the CellProfiler image analysis pipeline to quantify the cellular fluorescence intensity using the cytoplasmic mask (Fig. 6B, Table S3) and calculated the integrated cytoplasmic intensity of Bodipy558/568 fluorescence/cell (Fig. 6C). We noted that under these conditions there was little detectable cytosolic fatty acid but significant Bodipy558/568 C_12_ fluorescence was incorporated into reticular intracellular membranes, particularly in the perinuclear area of the cell (Fig. 6B). At later time points small punctate dots were also detected, predicted to be nascent LDs (results not shown). In both the control and the heterofibrin A1-treated cells there was a rapid initial accumulation of Bodipy558/568 C_12_ that reached a plateau at ∼10 min. However, in the presence of heterofibrin A1 both the initial rate of fatty acid accumulation and the maximal levels of fatty acids in the cell were significantly reduced consistent with a partial inhibition of uptake of the fluorescent fatty acid analogue.

Previous studies have shown that fluorescently labelled fatty acids can be metabolised by mammalian cells, albeit with altered characteristics to radiolabelled fatty acids [21], [22]. As some fluorescent fatty acid was taken up by cells pre-treated with heterofibrin A1, we used Bodipy558/568 C_12_ to further examine the metabolism of this labelled fatty acid in A431 cells in the presence or absence of heterofibrin A1. A431 cells were incubated ± varying concentrations of heterofibrin A1 or triacsin C overnight and subsequently incubated in 50 µM Bodipy558/568 C_12_ for 2 h. At the end of the incubation period the cells were harvested, the neutral lipids extracted and subsequently resolved by thin-layer chromatography (TLC) (Fig. 6D). Metabolites of Bodipy558/568 C_12_ were detected by their intrinsic fluorescence. Total neutral lipids were detected by subsequently staining the plates with primuline. Analysis of the TLC revealed several intriguing observations. First, despite the inhibition of fluorescent fatty acid accumulation in intracellular membranes by heterofibrin A1, there was only a small decrease detected in the total amount of Bodipy558/568 C_12_ in the cells. Second, examination of Bodipy558/568 C_12_ fluorescence revealed the accumulation of fluorescent metabolites in the heterofibrin A1-treated cells, resolving between the free cholesterol and free Bodipy558/568 C_12_ bands (Rf = 0.23 and 0.19), that were not detected in the control cells or in cells treated with triacsin C. A third band was resolved just above the free cholesterol band in control cells and in heterofibrin A1-treated cells, but not in triacsin C-treated cells suggesting the generation of an acyl-CoA species. Interestingly, in cells treated with 50 µM heterofibrin A1 the intensity of this band was reduced.

**Figure 7:**
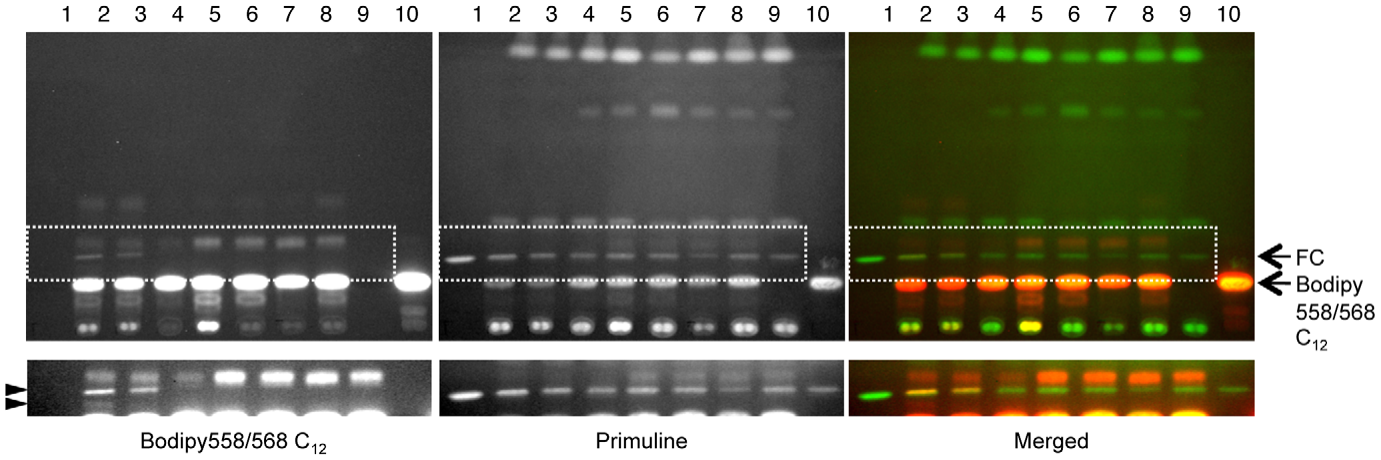
Effect of inhibitors of lipolysis and neutral lipid synthesis on fluorescent fatty acid metabolism: comparison to heterofibrin A1. A431 cells were incubated overnight in delipidated FCS supplemented with either heterofibrin A1 or known inhibitors of neutral lipid metabolic pathways, then supplemented with 50 µM Bodipy558/568 C_12_ for 2 h (lanes 2–9). Neutral lipids were extracted and resolved by TLC. Bodipy558/568 C_12_ was detected using its intrinsic fluorescence and total lipids detected using primuline. Lanes are as follows: (1) cholesterol standard, 10 µg, (2) 50 µM heterofibrin A1, (3) 20 µM heterofibrin A1, (4) 1 µM triacsin C, (5) 50 µM Orlistat, (6) 100 µM E600, (7) 600 µM 2-bromo octanoate, (8) Bodipy558/568 C_12_ 2 h, (9) Control cells, (10) Bodipy558/568 C_12_ control. Arrows (right) indicate free cholesterol (FC) and Bodipy558/568 C_12_. Arrowheads (left) indicate Bodipy558/568 C_12_ metabolites detected in the heterofibrin A1 treated cells only. Lower panels highlight regions of the TLC plate incorporating novel metabolites following prolonged exposure.

**Figure 8:**
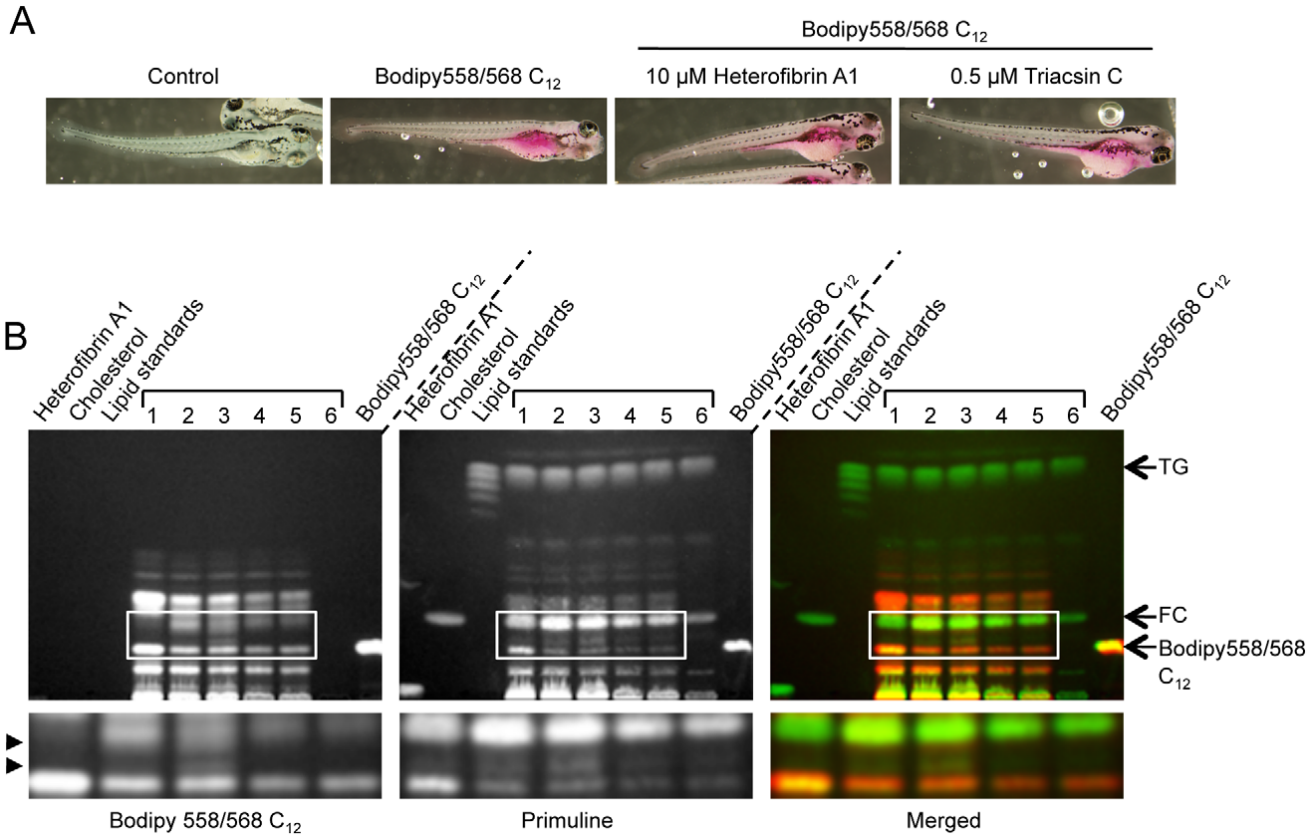
Metabolism of fluorescent fatty acid in zebrafish embryos. Zebrafish embryos (day 4–6 post-fertilisation) were incubated in the presence or absence of 10 µM heterofibrin A1 or 0.5 µM triacsin C for 2 h prior to the addition of Bodipy558/568 C_12_ for 6 h. Control embryos were incubated with vehicle (DMSO) only. Embryos were subsequently processed for fluorescence microscopy or TLC. (A) Embryos were fixed and fluorescent fatty acid imaged using a stereo dissecting microscope. (B) Embryos were homogenised, neutral lipids extracted and resolved using TLC. Total lipids were visualised using primuline and metabolised Bodipy558/568 C_12_ was detected by intrinsic fluorescence. The treatments were as follows: Lane 1, 0.5 µM triacsin C + Bodipy 558/568 C_12_; lane 2, 5 µM heterofibrin A1 + Bodipy 558/568 C_12_; lane 3, 10 µM heterofibrin A1 + Bodipy 558/568 C_12_; lane 4, Bodipy 558/568 C_12_; lane 5, DMSO + Bodipy 558/568 C_12_; lane 6 no treatment. Control lanes contained 65 µg heterofibrin A1, 10 µg cholesterol, 45 µg lipid standards and 24 µg Bodipy558/568 C_12_. Arrows delineate triglycerides (TG), free cholesterol (FC) and Bodipy558/568 C_12_. Arrowheads indicate Bodipy558/568 C_12_ metabolites accumulated in embryos in the presence of heterofibrin A1. Lower panels highlight regions of the TLC plate incorporating novel metabolites.

While analysis of radiolabelled oleic acid metabolism had suggested that heterofibrin A1 did not directly affect enzymes involved in TG biosynthesis, fluorescent fatty acid was both internalised and metabolised in the presence of heterofibrin A1 albeit with altered kinetics and products. As the identification of fluorescence fatty acid metabolites by TLC was problematic, we examined the possibility that heterofibrin A1 directly inhibits enzymes involved in either the neutral lipid biosynthetic pathway (diacylglycerol acyltransferase, DGAT) or lipolysis pathway by comparing the metabolism of Bodipy558/568 C_12_ in the presence of heterofibrin A1 to that of a known inhibitor of DGAT, 2-bromooctanoate [23], [24], and to two pan-lipase inhibitors, E600 and orlistat [25]–[27] (Fig. 7). Consistent with the data shown in Fig. 6D, heterofibrin A1 treatment resulted in the generation of unique Bodipy558/568 C_12_ metabolites resolving at the level of cholesterol, that were not detected following treatment with any of the other inhibitors. In contrast, the Bodipy558/568 C_12_ metabolite resolving above the cholesterol band was readily detectable in control cells, and in the presence of lipase or DGAT inhibitors, but was significantly reduced in heterofibrin A-treated cells and almost absent from triacsin C-treated cells. As heterofibrin A1 itself resolved higher up the silica plate with a Rf = 0.42 and was not intrinsically fluorescent at the wavelength used to detect Bodipy558/568 it is unlikely that these novel metabolites are derived from the esterification of heterofibrin A1 (see Fig. 8B). Theses data show that the novel metabolites of Bodipy558/568 C_12_ detected with heterofibrin A1 are unlikely to result from an effect on either lipases or DGAT. However, alterations in the metabolic processing of Bodipy558/568 C_12_ in the presence of heterofibrin A1 is at least partially similar to triacsin C, suggesting that heterofibrin A1 could also affect the formation of acyl-CoA.

### Effect of heterofibrin A1 on fluorescent fatty acid uptake and metabolism in zebrafish embryos

Finally to examine the effects of heterofibrin A1 on a model organism we used embryos of the zebrafish, *Danio rerio*. The zebrafish embryonic intestine is metabolically active [28] and can absorb and metabolise fluorescently labelled fatty acids albeit with varying degrees of efficiency [29]. We incubated embryos between days 4–6 post-fertilisation with 5–10 µM heterofibrin A1 or 0.5 µM triacsin C for 2 h prior to supplementation of the media with 10 µM Bodipy558/568 C_12_ for 6 h. Fixed embryos were subsequently examined by light microscopy. In contrast to control embryos, embryos incubated in Bodipy558/568 C_12_ contained significant fluorescence throughout the embryo but concentrated around the gut region consistent with the ingestion of fluorescent fatty acid (Fig. 8A). There was no detectable difference in fluorescence intensity or distribution when the embryos were pre-incubated with heterofibrin A1 (10 µM), triacsin C (0.5 µM) or vehicle control (DMSO, results not shown). To investigate the effect of heterofibrin A1 on the metabolism of Bodipy558/568 C_12_ in the zebrafish, we extracted neutral lipids from control or treated embryos and analysed them by TLC. Consistent with the results of Bodipy558/568 C_12_ metabolism in A431 cells, there was a similar accumulation of unusual metabolites in the presence of heterofibrin A1 in the body of the fish. These resolved with a similar mobility to the metabolites detected in the A431 cells, between the Bodipy558/568 C_12_ band and free cholesterol. These bands were not detected when the embryos was incubated in 0.5 µM triacsin C or with vehicle alone (DMSO) (Fig. 8B). While the nature of these metabolites is currently unknown, the results of the Bodipy558/568 C_12_ analysis suggest that a similar process is affected by heterofibrin A1 treatment in both cultured cells and in a model organism.

## Discussion

The aim of this study was to develop a cell-based high throughput assay for LD formation in order to screen a library of *n*-butanol extracts of Australian marine organisms for compounds with the ability to increase or decrease LD accumulation. While previous studies have identified small molecule inhibitors of LD formation in macrophage cell lines [6]–[11] and affecting the differentiation of adipocytes [5] we are unaware of any screens utilising Australian natural products. The Australian marine environment is a rich source of biodiversity, a fraction of which is held in the natural extracts collection at the Institute for Molecular Bioscience at The University of Queensland. We have utilised this extract collection to show that a relatively simple assay in a model cell line can be used to identify inhibitory molecules with conserved functional activity in less tractable cell lines and *in vivo* in a model organism. Furthermore, by using sub-fractionation and HPLC-MS we have been able to isolate specific bioactive molecules from relatively crude *n*-butanol extracts. While we have focussed in the current study on the heterofibrin family of molecules isolated from *Spongia (Heterofibria*) sp., bioactivity was also identified in a number of other marine extracts. Further analyses of these extracts are predicted to yield more novel molecular species with activity against LD formation and neutral lipid accumulation (Table 1).

The heterofibrin family of molecules isolated from *Spongia (Heterofibria)* sp. are novel diyne-ene fatty acids with either a carboxylic acid head group or the related monolactyl or dilactyl ester (Fig. 3A; [15]). As only the carboxylic acid heterofibrins A1 and B1 significantly inhibit LD formation in A431 cells, it is likely that the structure of the headgroup is important in the biological activity. Conjugated diyne-ene lipids are relatively rare among marine metabolites and are more common in the terrestrial natural products literature. Exocarpic acid, isolated from several plants of the family Santalaceae and Olacaceae [30] is a regioisomer of heterofibrin A1. While exocarpic acid has yet to be attributed biological activity, it is noteworthy that the related plant metabolite xymenynic acid has been reported to inhibit lipoxygenase and prostaglandin synthetase [31] and modify the fatty acid composition of mouse adipose tissue [32]. Further analysis of the heterofibrins will aid in identification of the cellular targets of these molecules.

LDs are ubiquitous organelles that play a crucial role in maintaining the cellular levels of lipids by regulating the interplay between lipid storage and release [1], [2]. Changes in LD formation and catabolism are associated with a wide range of metabolic disorders, genetic diseases and pathogen infection. Excessive LD accumulation is a hallmark of some of the most prevalent contemporary metabolic disorders including obesity [33], hepatic steatosis [19] and atherosclerosis [34]. As traditional dietary approaches are having little effect on controlling weight gain, there is increased interest in understanding the molecular regulation of triglyceride storage in order to identify pharmaceutical targets for controlling lipid storage and handling by cells [35]. In addition to the physiological importance of regulating LDs in lipid metabolism, pathogens such as members of the *Flaviviridae* family of positive strand RNA viruses, including hepatitis C virus and Dengue virus, induce LD formation and utilise LDs for viral assembly [20], [36]. Despite the very obvious importance of LDs in many aspects of human disease, fundamental questions regarding their cell biology remain unanswered. Identifying small molecules with bioactivity affecting LD formation or catabolism will aid in our understanding of the cellular processes underlying a variety of pathophysiological disorders. While studies aimed at identifying the cellular target(s) of heterofibrin A1 are ongoing, the unique fluorescent fatty acid metabolites generated suggest a mechanism of action distinct to inhibition of neutral lipid lipases or DGAT. Furthermore, partial similarity to triacsin C suggests that heterofibrin A1 may have more than one intracellular target. Analyses of the LD proteome (reviewed in [37]) and RNAi-based screening in yeast [38]–[40] and drosophila [41] have demonstrated that the regulation of LDs is highly integrated into other cell biological processes including membrane trafficking, cell signalling and metabolic pathways. Historically the use of small molecule inhibitors has proven invaluable in dissecting these cell biological processes. The identification of small molecules with bioactivity targeting regulatory proteins in the LD biogenesis and catabolism pathways will further understanding of this important cellular organelle.

## Supporting Information

Table S1 Lipid droplet analysis pipeline.
(DOC)

Table S2 Cytotoxicity image analysis pipeline.
(DOC)

Table S3 Cytoplasmic analysis pipeline.
(DOC)
