# Supplementary material for: High-Throughput Screening of Australian Marine Organism Extracts for Bioactive Molecules Affecting the Cellular Storage of Neutral Lipids
Source: PLoS One. 2011 Aug 8;6(8):e22868. doi: 10.1371/journal.pone.0022868 (PMC3152550; doi:10.1371/journal.pone.0022868)
Supplement: Table S1 — Lipid droplet analysis pipeline. (DOC) [file pone.0022868.s001.doc]

**Table S1: Lipid droplet analysis pipeline**

| **Module** | **Function** |
| --- | --- |
| LoadImages | Load image sets into pipeline |
| RescaleIntensity | Rescales intensity of native nuclei image from 0-1 |
| RescaleIntensity | Rescales intensity of native lipid droplet image from 0-1 |
| CorrectIlluminationCalculate | Calculates and produces a rolling ball background correction image of rescaled lipid droplet image |
| CorrectIlluminationApply | Subtracts correct illumination image from rescaled lipid droplet image |
| IdentifyPrimaryObjects | Identifies nuclei within images based upon intensity and size (representative values: min 0.2, max 1.0) |
| IdentifySecondaryObjects | Identifies cytosolic areas using nuclei as a parent object and lipid droplet background staining to identify edges |
| IdentifyPrimaryObjects | Identifies lipid droplets within images based upon intensity and size (representative values: min 0.14, max 1.0) |
| Relate | Associates ‘children’ objects with ‘parent’ objects |
| ExportToExcel | Produces excel spreadsheets containing statistical information analysis |

Automated analysis of the number of lipid droplets per cell used the listed modules within the CellProfiler software. Using this pipeline we were able to identify nuclei, identify the cytosolic area of each cell via propagation from individual identified nuclei using Bodipy 493/503 cytosolic fluorescence to define the edge of a secondary object (the cell), identify lipid droplets and directly relate specific droplets to specific cells. It should be noted that in cells lines in which LDs are observed to cluster the accuracy of the LD analysis pipeline is reduced.
