# Supplementary material for: High-Throughput Screening of Australian Marine Organism Extracts for Bioactive Molecules Affecting the Cellular Storage of Neutral Lipids
Source: PLoS One. 2011 Aug 8;6(8):e22868. doi: 10.1371/journal.pone.0022868 (PMC3152550; doi:10.1371/journal.pone.0022868)
Supplement: Table S2 — Cytotoxicity image analysis pipeline. (DOC) [file pone.0022868.s002.doc]

**Table S2**: Cytotoxicity image analysis pipeline

| **Module** | **Function** |
| --- | --- |
| LoadImages | Load image sets into pipeline |
| RescaleIntensity | Rescales intensity of native image from 0-1 |
| CorrectIlluminationCalculate | Calculates and produces a rolling ball background correction image of rescaled image |
| CorrectIlluminationApply | Subtracts correct illumination image from rescaled image |
| IdentifyPrimAutomatic | Identifies objects within images based upon intensity and size (representative values: min 0.1, max 1.0) |
| ExportToExcel | Produces excel spreadsheets containing statistical information analysis |

Automated analysis of nuclei number was determined as a surrogate for cytotoxicity using the listed modules within the CellProfiler software.
