# Supplementary material for: High-Throughput Screening of Australian Marine Organism Extracts for Bioactive Molecules Affecting the Cellular Storage of Neutral Lipids
Source: PLoS One. 2011 Aug 8;6(8):e22868. doi: 10.1371/journal.pone.0022868 (PMC3152550; doi:10.1371/journal.pone.0022868)
Supplement: Table S3 — Cytoplasmic analysis pipeline. (DOC) [file pone.0022868.s003.doc]

**Table S3: Cytoplasmic analysis pipeline**

| **Module** | **Function** |
| --- | --- |
| LoadImages | Load image sets into pipeline |
| RescaleIntensity | Rescales intensity of native nuclei image from 0-1 |
| RescaleIntensity | Rescales intensity of native cytoplasmic image from 0-1 |
| IdentifyPrimaryObjects | Identifies nuclei within images based upon intensity and size |
| IdentifySecondaryObjects | Identifies cytoplasmic areas using nuclei as parent objects and background staining to identify edges |
| MeasureObjectIntensity | Measures identified objects fluorescence intensity |
| ExportToExcel | Produces excel spreadsheets containing statistical information analysis |

Automated analysis of the integrated cytoplasmic intensity per cell used the detailed modules within the Cell Profiler software. Using this pipeline we were able to identify nuclei, identify the cytosolic area of each cell via propagation from individual identified nuclei using Bodipy558/568 C_12_ cytosolic fluorescence, to define the edge of a secondary object (the cell) and measure the integrated cytoplasmic intensity of Bodipy558/568 fluorescence per cell.
